# Infantile tremor syndrome: a case series from the tertiary care center of Nepal

**DOI:** 10.3389/fnut.2026.1783728

**Published:** 2026-06-23

**Authors:** Srijana Basnet, Luna Bajracharya, Sudeep K.C

**Affiliations:** Department of Pediatrics, Tribhuvan University Teaching Hospital, Institute of Medicine, Kathmandu, Nepal

**Keywords:** pallor, skin changes, tremor, vegetarian, vitamin B12

## Abstract

Infantile tremor syndrome (ITS) is a pediatric disorder characterized by tremors, developmental regression, pallor, and hyperpigmentation, which is frequently associated with vitamin B12 deficiencies. Despite being recognized for decades, it remains underdiagnosed and lacks standardized management guidelines. We describe a case series of children with clinical characteristics suggestive of ITS. All children included in this study were from strictly vegetarian families and exhibited tremors, pallor, and characteristic skin changes. Four of them were diagnosed with ITS after recovering from their illnesses while in the pediatric intensive care unit (PICU). Investigations found that all cases were vitamin B12 deficient. Three of our cases had cortical atrophy on neuroimaging., with two patients requiring blood transfusions despite the prevalence of neurological symptoms. Biochemical tests confirmed vitamin B12 deficiency. All infants were treated with dietary changes, multivitamin and mineral supplements, and vitamin B12 therapy. Significant clinical improvement was observed following the intervention. This case series highlights the variable dermatological, hematological, and neurological presentations of ITS, emphasizing the importance of early recognition and treatment, including maternal therapy.

## Introduction

Infantile tremor syndrome (ITS) is a clinical condition characterized by tremors, anemia, pigmentary changes, delays or regression in developmental milestones, and muscular hypotonia (1). Tremors and developmental regression are often the predominant clinical features. The syndrome is frequently misdiagnosed with seizure disorders or neurodegenerative disease, resulting in unnecessary diagnostic tests. A high index of suspicion, a history of a vegetarian diet in both mother and child, onset between 5 months and 5 years, and supportive laboratory findings confirm the diagnosis (2). This case series presents a variety of clinical presentations of infantile tremor syndrome.

## Case descriptions

### Case 1

An 8-month-old boy was referred to Tribhuvan University Teaching Hospital (TUTH) for severe respiratory distress while being treated for pneumonia. He required mechanical ventilation for the first few days. During recovery, anemia and skin hyperpigmentation were observed, as well as apathy, tremor, absent reflexes, and hypotonia. He was born at full term through spontaneous vaginal delivery at home, weighing 3,700 grams. He had a worldwide developmental delay and a developmental age of 4 months. The mother was a strict vegetarian, and the infant was exclusively breastfed prior to admission. The child was treated with 1 week of ceftriaxone for pneumonia, but his respiratory distress worsened, so he was referred to us. Anthropometry revealed a weight of 8.7 kg, a length of 71 cm, a head circumference of 41 cm, W/L: 0 to +1 SD, L/A: 0 SD, HC/A: −2SD to −3SD. The weight for length (W/L), length for age (L/A), and head circumference (HC/ A) for age were 0 to +1 SD, 0 SD, and −2SD to −3SD, respectively.

Further detailed examination also revealed that the child had inspiratory stridor. He had a generalized, coarse, distal tremor with no other types of abnormal movements. He had peripheral hypotonia without deep tendon reflexes. His muscle power in all groups was 4/5.

The patient’s hemoglobin level was 10.0 g/dL, total RBC count was 3.66 million/μL, MCV was 85.44 fL, and MCH was 27.67 pg. A peripheral smear showed a dimorphic image with macrocytic and normocytic normochromic cells. MRI showed diffuse cerebral and corpus callosum atrophy. Further laboratory evaluation revealed 25-hydroxyvitamin D of 23.3 ng/mL, vitamin B12 of 131 pg/mL, serum iron of 108 μg/dL, total iron-binding capacity (TIBC) of 272 μg/dL, and ferritin of 193 ng/mL.

Considering ITS, intramuscular vitamin B12 and propranolol were administered, resulting in gradual improvement within a week and the start of oral feeding. He also received vitamin D, iron, folic acid, and multivitamin supplements. The child was admitted to the ward, his oxygen was lowered, and he became aware, active, and playful, feeding orally. He then suffered new-onset tachypnea with a diffuse wheeze that responded to salbutamol; reliever and controller therapy were started, and he was discharged. The methylcobalamin injection dose was 1,000 mcg OD for 14 days, followed by 1,000 mcg/week for 6 weeks, and finally monthly until symptoms subsided. After 4 months of treatment, anemia was treated and the symptoms disappeared, therefore he was continued on oral supplementation for another 5 months.

### Case 2

A 12-month-old girl was taken to TUTH after experiencing abnormal movements of her left hand for 3 days. Medical records showed that she had been intubated for suspected recurring focal seizures. When she arrived, she was still intubated and had ongoing upper-limb tremors, mainly in her hands, so she was admitted to the pediatric intensive care unit (PICU). She was born via emergency lower-segment cesarean section with a birth weight of 3.2 kg and had an uneventful perinatal course. Developmentally, she could crawl but not stand without support (gross motor delay), had an immature pincer grasp (fine motor delay), and could wave “bye-bye.” Immunizations were up-to-date. Until 8 months, the infant was exclusively breastfed and consumed little supplemental food. The mother and the entire family were strict vegetarians. Anthropometry revealed a weight of 10 kg, a length of 71 cm, a head circumference of 45 cm, W/L: +2SD, L/A: −0 to −2 SD, and HC/A: 0SD. She exhibited significant perioral and limb tremors with hypotonia.

Upon evaluation, severe anemia (Hb 4.3 g/dL, MCV 91 fL, MCH 32.9 pg) was observed, with peripheral smear findings ranging from normocytic normochromic to microcytic hypochromic. CECT showed bilateral subdural hygromas, cerebral atrophy, and subtle gyriform hyperdensity in the right frontal lobe. Vitamin B12 levels were low in both the child (56 pg./mL) and the mother.

She had a blood transfusion for anemia and was gradually weaned from mechanical ventilation. Tremors were treated with oral propranolol, and 1000mcg of methylcobalamin was administered intramuscularly. The EEG, ophthalmologic, and hearing examinations were all normal. Physiotherapy was initiated. The child improved clinically and was discharged with a plan for neurodevelopmental monitoring and continued vitamin B12 treatment. Inj Methylcobalamin 1,000 mcg was administered once daily for 14 days, twice weekly for 2 weeks, once weekly for 3 months, and once monthly for 6 months. After 7 months of treatment, the child showed significant improvement in meeting previously acquired developmental milestones.

### Case 3

A 5-month-old boy was referred to TUTH for further evaluation after a 20-day history of decreased activity and atypical skin lesions. Over the previous 3 weeks, he had initially refusal to feed, followed by a decreased level of consciousness and an appearance of drowsiness. There had been two episodes of generalized tonic–clonic seizures 1 day before the presentation.

He had been treated at another center for suspected livedo reticularis, and investigations confirmed pancytopenia. He then experienced a 20–30 s. He was breastfed exclusively, and his mother was a strict vegetarian. Prior to the illness, he had achieved neck holding, bidexterous reach, social smile, and recognition of his mother; however, neck holding was not observed at our center. On examination, the child had hyperpigmented, macular, lacy reticular lesions involving the bilateral lower limbs, abdomen, and chest, gradually darkening and progressive. Pallor was moderate. The child had brown scalp hairs. Anthropometry revealed a weight of 7.5 kg, a length of 67 cm, a head circumference of 42 cm, W/L: 0 to −1SD, L/A: +1 to +2 SD, HC/A: 0 to −1SD. The child was lethargic. Muscle bulk was normal, but there was generalized hypotonia with diminished deep tendon reflexes.

Hemoglobin level was 10.2 g/dL, with a platelet count of 1,10,000/μL. The total leukocyte count was 5,500/μL (neutrophils 41% and lymphocytes 56%). Red cell indices were MCV 75.7 fL, MCH 24.9 pg., and MCHC 32.8 g/dL. Liver enzyme levels were high (AST 122 U/L, ALT 108 U/L, and ALP 256.78 U/L). MRI revealed mild cerebral atrophy affecting the corpus callosum, while EEG suggested possible epileptic discharges in the right occipital region. Serum vitamin B12 levels were significantly lowered (<83 pg./mL). So intramuscular vitamin B12 1000mcg/day was initiated and administered weekly for 2 weeks. During the treatment, we detected a generalized, coarse tremor in his upper and lower limbs, as well as his head. The child was then diagnosed with ITS. He was then given vitamin B12 twice a week for 2 more weeks, then once a week for 3 months, and finally once a month for 3 months. Though the child’s tremor worsened with the initiation of vitamin B12 supplementation, he gradually improved after increasing the dose and receiving oral levetiracetam at a dose of 20 mg/kg/day. His appetite and sensorium gradually improved. The child was also investigated for other possible causes of seizures. The ABG was normal. Urine for the metabolic screening was normal. After 4 months of treatment, his symptoms improved, and his anemia was corrected.

### Case 4

A 9-month-old girl was referred to TUTH due to increased respiratory distress and a possible need for mechanical ventilation. She had been suffering from noisy breathing since she was 6 months of age and receiving treatment at a local hospital. Two weeks before admission at our center, the child had tachypnea with severe retraction, and the he was hospitalized to another center’s ICU with BiPAP support. The infant was breastfed exclusively until 9 months old, with no introduction of complementary feeds. Her parents were strictly vegetarians. An anthropometric examination revealed a weight of 6 kg, length of 65 cm, head circumference of 42 cm, W/L: −2SD, L/A: −2 SD, and HC/A: −1SD to −2SD.

She had sparse brown scalp hair, hypotonia, and hyperpigmentation on her knuckles.

Hematological evaluation showed anemia (hemoglobin 5.8 g/dL, TLC 26200/μL, platelet count 195,000/μL at nadir). Liver enzymes were elevated (AST 148 U/L, ALT 118 U/L, ALP 272 U/L), with hypoalbuminemia (albumin 3.6 g/dL). Vitamin B12 level was markedly reduced at 23 pg./mL.

She was admitted with the diagnosis of pneumonia and was ventilated for 12 days, then gradually weaned to bubble CPAP. Given vitamin B12 deficiency, she was started on IM Inj Vitamin B12 once weekly along with IV antibiotics. She also received other vitamin supplements. On the subsequent days, she was noticed having generalized coarse tremor involving the upper and lower limbs and head after the initiation of vitamin B12 injection. Considering ITS, dose of vitamin B12 increased to 1,000 mcg once daily for 14 days, once weekly for 4 weeks, then once per month for 6 months.

She was managed with broad-spectrum antibiotics, supportive care, repeated PRBC and platelet transfusions. ENT evaluation was also performed, and she was found to have laryngomalacia and planned to undergo supraglottoplasty. After she was extubated, she was kept on CPAP and gradually weaned off to room air. The child was investigated for other causes of tremor as well. ABG was normal. Urine for the metabolic screen was normal. By discharge after 1 month of treatment, tremors disappeared, and blood parameters improved. Her mother was also treated with Vitamin B12 supplementation.

### Case 5

A 15-month-old boy was admitted to the PICU for management of hypernatremic dehydration and sepsis. During treatment, he developed a focal seizure affecting the left upper limb, appeared pale, and showed hyperpigmentation of the knuckles along with tremors. Further history revealed exclusive breastfeeding until 8 months and a strictly vegetarian mother and family. He also had global developmental delay. Anthropometric examination revealed a weight of 11.7 kg, a length of 79 cm, a head circumference of 45 cm, W/A: +2SD, W/L: −1 to −2 SD, L/A: 0 to +2SD, and HC/A: −1SD to −2SD.

After recovery from sepsis, he was noticed having generalized, coarse tremor involving upper and lower limbs that appears when awake and disappears during sleep. His muscle bulk is normal in all limbs. He had generalized hypotonia with muscle power of 4/5 across all muscle group in the upper and lower limbs. Deep tendon reflexes were exaggerated. The laboratory results showed hemoglobin 7.5 g/dL, total leukocyte count 4,900/μl, platelet count 65,000/μl, and peripheral smear with normocytic normochromic red cells, few macrocytic hypochromic cells, and polychromasia. Serum iron was 174 μg/dL, TIBC was 197 μg/dL, and ferritin was 119 ng/mL. Vitamin B12 was low at 60 pg./mL. His EEG showed epileptic potential over the right parietotemporal region, but his MRI head was normal. Management included injectable vitamin B12 supplementation daily for 2 weeks in addition to the correction of hypernatremic dehydration, seizure, and sepsis. Following 2 weeks of treatment, the patient showed marked clinical improvement and was discharged on inj 1,000 mcg/week for 6 weeks after which all symptoms disappeared, and anemia was corrected. The patient was then continued on oral vitamin B12 for 3 months.

## Discussion

There is a paucity of data on ITS from Nepal (3). However, there are several case reports of ITS from different parts of India (2, 4). The exact incidence of ITS worldwide is not known, but in India, it accounts for 0.2–2% pediatric hospital admissions (4, 5).

In this case series, children were between 5 and15 months, and the mothers of all children were strict vegetarians. Tremor was the most noticeable sign. Tremors were predominantly observed in the facial, labial, lingual, and laryngeal muscles, often presenting as asymmetric or multifocal. Most of these children were either developmentally delayed or lost the previously achieved milestones along with tremor.

Four of the five patients in this case series were quite sick, requiring PICU admission and mechanical ventilation for respiratory failure. In two of the cases, tremor was absent at admission but became apparent later during the course of illness. It is suggested that concurrent respiratory or gastrointestinal infections frequently precipitate acute neurological decline, leading to the onset of tremors (2, 6, 7). Tremors are mostly due to structural and functional alterations of the extrapyramidal system (1). This case series highlights that ITS is frequently identified incidentally during the management of another critical condition and may remain unrecognized in its early stages.

Few other studies have reported worsening tremor following initiation of vitamin B12 therapy, as in two of our cases (2, 8–10). This phenomenon is thought to reflect a paradoxical effect of vitamin B12 in severe deficiency, possibly due to a rapid shift in cerebral metabolism triggering tremor (11, 12). It has also been suggested that this may result from persistent hyperglycinemia or from a hypersensitization effect associated with vitamin B12 deficiency, involving endothelial dysfunction mediated by excitatory amino acids, along with increased levels of methyltetrahydrofolate and homocysteine (13, 14). Tremor improved with ongoing vitamin B12 therapy, though propranolol was also needed to manage it. The intensity of tremor and the recovery time differed among patients, consistent with findings from other studies (15).

There have always been overlapping features of tremor and seizure (16). Tremor might be confused as a seizure or a seizure could be the manifestation of Vitamin B12 deficiency (15, 17). We treated patients who had seizures with EEG changes.

**Figure 1:**
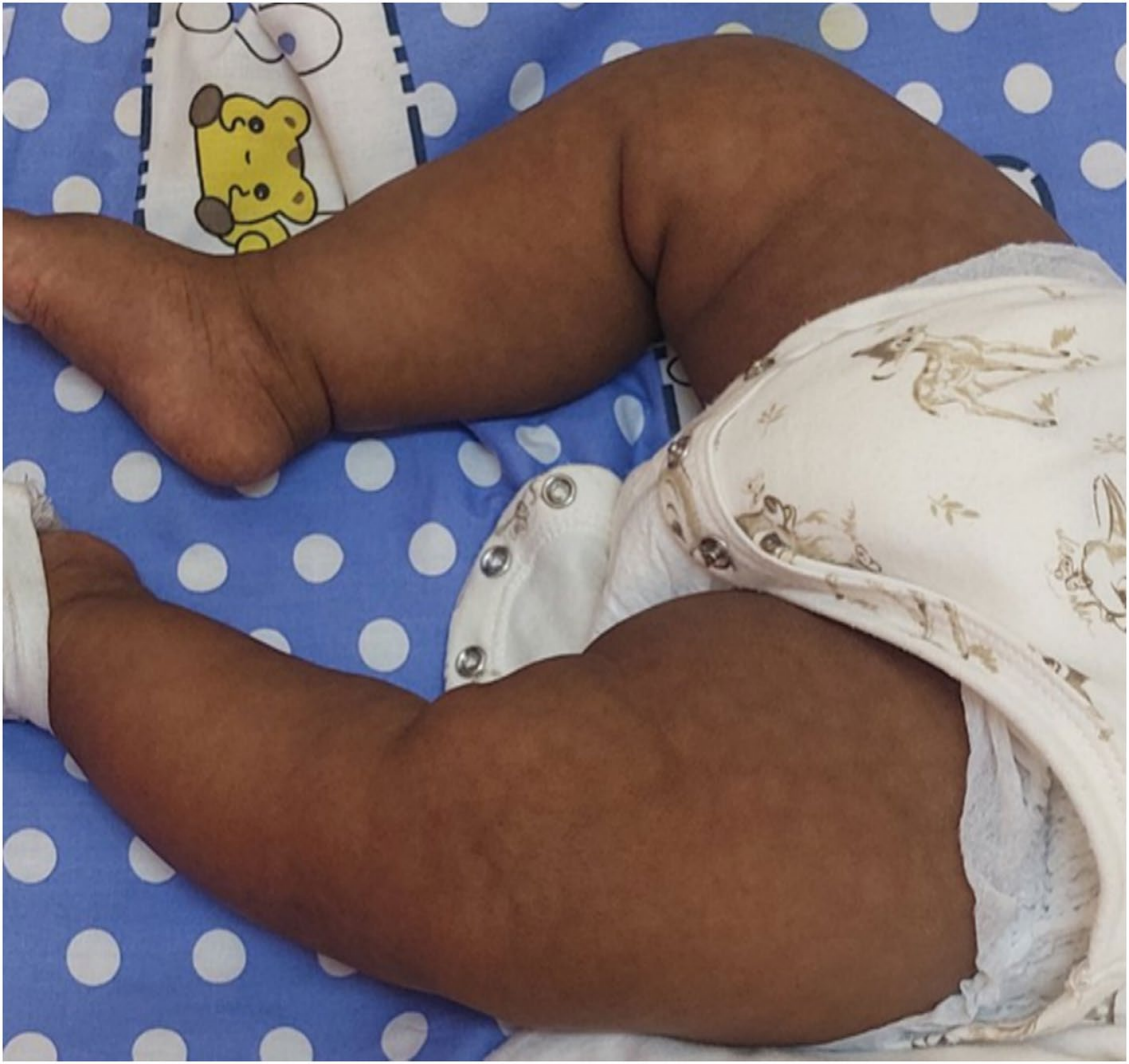
Diffuse honeycomb or reticulate hyperpigmentation pattern over bilateral lower limbs.

It is well established that neurological signs of vitamin B12 deficiency may occur in the absence of hematological abnormalities, a condition known as hematological–neurological dissociation (18). However, in this case series, all children were anemic (hemoglobin <11 g/dL), with two presenting severe anemia that required blood transfusion. The most common skin changes were pallor and knuckle hyperpigmentation. Notably, one child exhibited a diffuse honeycomb or reticulate hyperpigmentation pattern involving both the trunk and limbs (Figure 1), similar to the changes described by Goraya et al.

No standardized guidelines exist for the management of ITS. Most authors have managed affected infants symptomatically using various combinations of vitamin B12 supplementation, dietary modifications, multivitamins, and mineral supplements for weeks to months (11). In this case series, there is slight variation in the dose and duration of Vitamin B12. MRI in three patients showed cortical atrophy, but all children in our case series regained developmental milestones, along with alertness, interest in their surroundings, and hematological improvement after treatment.

Although MRI was not conducted in all cases, cerebral atrophy was the most observed finding. A right frontal hyperintensity suggestive of a lesion was noted in one case, similar to the finding reported by Thora S. et al. (19). Repeat MRI was not performed in our patients; however, reversal of cerebral atrophy following treatment has been described in the literature. Delayed diagnosis can result in permanent neurological deficits despite therapy, highlighting the need for a high index of suspicion and early identification (20).

ITS remains a significant and prevalent condition in developing countries, particularly among populations with low-socioeconomic status. There are a few limitations of this study. Alternative diagnoses were not comprehensively excluded, as not all relevant investigations were performed. Due to financial constraints, MRI, CSF analysis, and bone marrow examinations were not performed in all patients, with most tests being paid out of pocket by families. For the same reason, repeat MRI and follow-up vitamin B12 assessments were also not performed. The exact timeline for improvement in clinical manifestations has also not been documented. Given the limited existing literature, this case series still contributes additional clinical evidence and insights to the scientific community.

## Conclusion

Infantile tremor syndrome should be considered in young children presenting with tremors and developmental regression, particularly in those from vegetarian families. Early recognition and vitamin B12 supplementation can lead to rapid clinical improvement and prevent long-term neurological deficits.

## Data Availability

The original contributions presented in the study are included in the article/supplementary material, further inquiries can be directed to the corresponding author.
